# 

**DOI:** 10.1192/bjb.2025.22

**Published:** 2026-02

**Authors:** Savva Pronin

**Affiliations:** Specialty Trainee in Child and Adolescent Psychiatry, Tavistock and Portman NHS Foundation Trust, London, UK.

**Figure:**
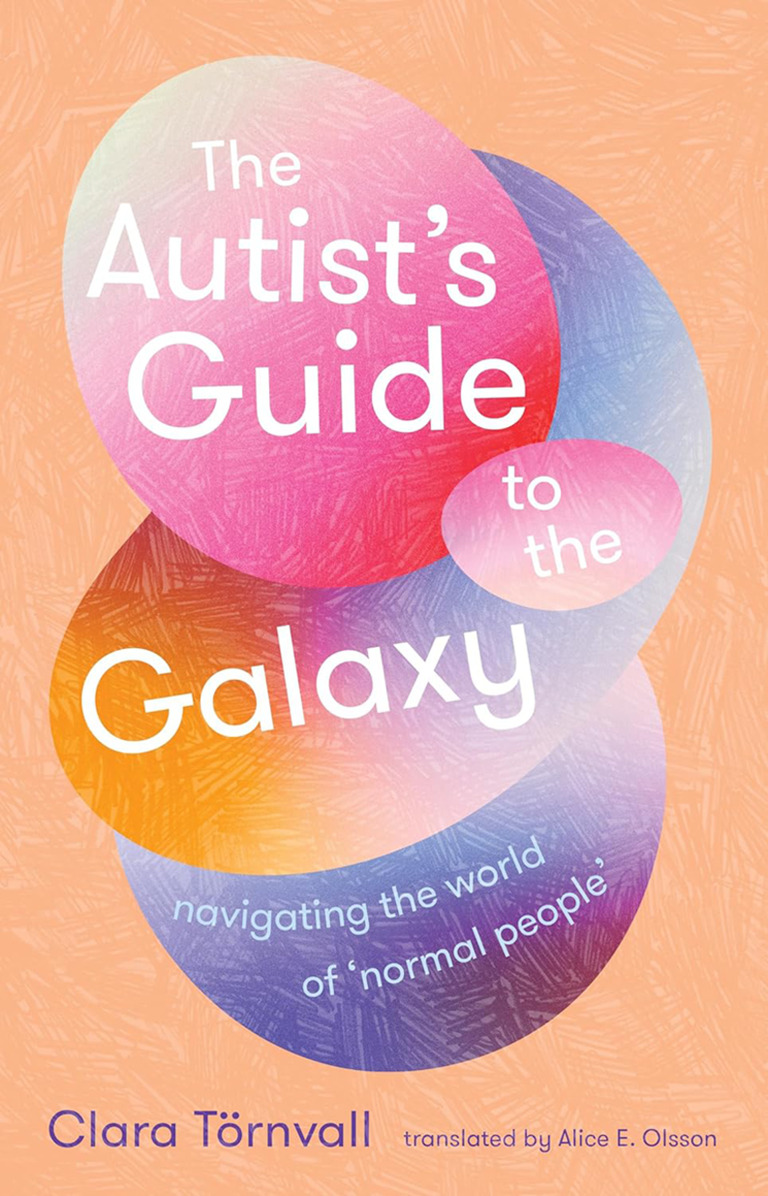

Clara Törnvall’s book flips the traditional perspective on autism. Instead of another book by neurotypicals about autistic people, this is a book by autistic people for autistic people – serving as a guide to the often bewildering world of neurotypicals. Translated into English by Alice E. Olsson and interspersed with illustrations by Anneli Furmark, the book is both accessible and appealing.

Törnvall, a journalist and producer, structures her second book as a guidebook that covers a varied mix of topics, from social greetings and dating to education and workplace interactions, in a varied mix of formats, including glossaries, summary sections and a collection of first-hand experiences from autist contributors adding a phenomenological depth. Notably absent is a neurotypical perspective, which could have provided additional balance. However, this absence itself is thought-provoking – mirroring how autistic voices are often missing in mainstream discourse.

Törnvall’s writing is direct, accessible and well suited to a younger audience. She keeps things contemporary with references to TV shows and online spaces like Reddit, making the content feel relevant and relatable. At times the book can feel loosely structured and repetitive, but the short chapters make it a fast read. A bibliography with suggested further readings is included, although some references lack citations, making certain claims feel more anecdotal than academic.

Although the tone superficially appears lighthearted or playful, Törnvall conveys a strong sense of frustration – an unfiltered honesty about the struggles faced by autistic individuals in a majority-neurotypical world. She highlights the blind spots in how neurotypicals view autism, particularly their reliance on non-verbal cues and the underappreciated impact of sensory differences. For neurotypical readers, this book offers plenty to reflect on and, for clinicians, it may prompt consideration of how they communicate with autistic individuals.

While the book risks overgeneralisation of both autistic and neurotypical experiences, and one needs to be mindful of the Western-centric perspective, it may be a good starting point for young autistic people grappling with their diagnosis. It may also be insightful for family members and friends seeking to better understand an autistic person’s inner world. Ultimately, The Autist’s Guide to the Galaxy is a personal and emotive inversion of the usual narrative – offering autistic readers a guide to navigating a world not built for them.

